# Cellulose-Based Polymer Blends for Oral Mucoadhesion: Impact of Hydration and Surface Interactions

**DOI:** 10.3390/polym18101227

**Published:** 2026-05-17

**Authors:** Monika Rojewska, Emilia Jakubowska, Klaudia Szelejewska, Maja Nowaczyk, Anna Froelich, Krystyna Prochaska, Tomasz Osmałek

**Affiliations:** 1 Institute of Chemical Technology and Engineering, Faculty of Chemical Technology, Poznan University of Technology, ul. Berdychowo 4, 60-965 Poznan, Poland; maja.nowaczyk@student.put.poznan.pl; 2 Department of Pharmaceutical Technology, Faculty of Pharmacy, Poznan University of Medical Sciences, 3 Rokietnicka, 60-806 Poznan, Poland; ejakubowska@ump.edu.pl (E.J.); kkruger@ump.edu.pl (K.S.); froelich@ump.edu.pl (A.F.); tosmalek@ump.edu.pl (T.O.)

**Keywords:** hydroxypropyl methylcellulose, mucoadhesive polymer blends, wettability, surface free energy, mucoadhesive strength, swelling

## Abstract

Hydration, interfacial interactions, and matrix stability are critical determinants of the mucoadhesive behavior of cellulose-based polymers. In this study, we investigated the physicochemical and mucoadhesive behavior of hydroxypropyl methylcellulose (HPMC), Carbopol 974P NF, and Kollidon VA 64, along with their binary blends (1:1, *w*/*w*) in the context of oral mucosal drug delivery. Wettability, surface free energy, mucoadhesion, and hydration-induced morphological changes were systematically evaluated using contact angle measurements, adhesion and water uptake studies, and real-time surface dissolution imaging (SDi2). The investigated systems displayed markedly different water contact angles: HPMC 103.4 ± 2.7°, Carbopol 47.2 ± 2.3°, Kollidon 36.0 ± 1.8°, HPMC:Carbopol 51.3 ± 2.8°, and HPMC:Kollidon 53.9 ± 3.4°. The corresponding surface free energy (SFE) values ranged from 12.0 mJ/m^2^ for HPMC to 70.5 mJ/m^2^ for Kollidon. Experiments were performed under saliva-mimicking conditions containing 0.1% (*w*/*v*) mucin. The HPMC:Carbopol blend exhibited superior mucoadhesive performance and mechanical stability compared with HPMC alone or with the HPMC:Kollidon blends. In 2% (*w*/*v*) mucin, the HPMC:Carbopol blend reached a mucoadhesive force of approximately 1.35 N, whereas HPMC and HPMC:Kollidon showed lower values of approximately 0.5–0.75 N and 0.60 N, respectively. After 96 h at 85% RH, the swelling index increased from 14.8 ± 0.5% for HPMC to 29.4 ± 0.3% for HPMC:Carbopol. The incorporation of Carbopol increased the polar contribution to the surface free energy of HPMC-based blends and promoted stable gel layer formation, whereas Kollidon-containing systems underwent rapid disintegration and asymmetric deformation. SDi2 imaging showed that the HPMC disk changed proportionally by approximately 18% in both height and width during 12 h, whereas the HPMC:Kollidon disk almost completely dissolved after approximately 6 h. These results demonstrate that rational selection and combination of cellulose-based polymers can be used to control hydration, interfacial properties, and mucoadhesion, with HPMC:Carbopol blends showing strong potential for oral mucosal drug delivery.

## 1. Introduction

Oral mucosal drug delivery is a promising strategy; however, it remains challenging despite extensive research [1,2,3,4]. Drug delivery through the buccal mucosa offers several advantages, including avoidance of the gastrointestinal tract and first-pass metabolism, but its effectiveness is influenced by multiple formulation- and tissue-related factors [5]. Mucoadhesive polymers can adhere to mucosal surfaces, prolong contact time and enable controlled interaction with the tissue [6,7]. Therefore, the development of effective mucoadhesive systems requires careful selection of polymers with balanced adhesion and mechanical properties [8,9]. For buccal application, tablets should be thin (≈1–2 mm) and small in diameter (typically 6–8 mm) to ensure patient comfort and minimize interference with oral functions [9].

The oral mucosa contains mucins, which are large glycoproteins that play a key role in polymer adhesion. Mucins constitute approximately 1–5% of saliva and form a protective mucus layer that is essential for lubrication, antimicrobial defense, and epithelial integrity [10,11,12,13,14]. Polymer–mucin adhesion involves physical entanglement and chemical interactions, including electrostatic interactions, hydrogen bonding, hydrophobic interactions, and van der Waals forces [15,16,17,18,19]. Mucoadhesion generally proceeds through three stages: wetting and swelling of the polymer, entanglement with mucin chains, and formation of weak chemical bonds [20,21]. High wettability can enhance polymer hydration, swelling, and retention at the mucosal surface.

Using blends of mucoadhesive polymers can improve the surface properties (wetting) of drug carriers, leading to improved performance over single-polymer formulations [3]. In our previous studies [22,23] we have shown that polymer blending can produce synergistic effects, in which the combined performance of the polymers exceeds that of the individual components. Such synergy may improve the wettability of a formulation and contribute to more effective drug release. Moreover, studies have shown that blends can enhance the resilience of matrix tablets against varying hydrodynamic conditions during dissolution [24]. Some tablets and buccal films both use four polymers, and the individual polymers provide various functionalities [25]. Polymer blends are also widely used in lubricating ophthalmic drops, where synergistic polymer combinations improve key product attributes, including lubrication, viscosity, and ocular retention time, e.g., blends including HPMC and Carbopol [24,26,27].

The present study is related to our previous investigations on the wettability, swelling, sorption, and surface free energy of mucoadhesive polymers and API-containing mucoadhesive systems; however, it addresses a distinct scientific question. Unlike our earlier studies in saliva or vaginal fluids, which focused mainly on Carbopol, Noveon AA-1, HEC, Kollidon, and fluconazole-containing systems [22,23], the present work isolates HPMC-based binary matrices and evaluates their behavior in mucin-containing simulated salivary media. The key added value of this study is the use of an API-free, mucin-relevant model that separates polymer–polymer and polymer–mucin contributions from drug-specific effects and directly examines whether favorable wettability and surface energetics translate into measurable mucoadhesion and long-term matrix integrity. The present work extends this research line by separating polymer-matrix effects from drug-specific effects and by evaluating hydration-mediated mucoadhesion under mucin-relevant oral conditions. This design allows the study to distinguish rapid wetting from functional mucoadhesion, which requires not only interfacial hydration but also polymer–mucin bonding and time-dependent matrix integrity.

Matrix tablets based on hydrogel-forming polymers are commonly used in sustained-release formulations. HPMC (hydroxypropylmethylcellulose) is one of the most widely used polymers for this purpose [28,29,30]. Literature reports indicate that HPMC of varying molecular weights can be blended to provide a practical strategy for adjusting drug release profiles [24]. Moreover, studies have demonstrated that mixed matrices with HPMC can effectively modulate the pH-dependent dissolution profiles of ionizable APIs [24,31]. Besides viscosity and gel strength, the incorporation of other polymers with HPMC can affect matrix hydration, as well as the rates of swelling and erosion [32,33,34]. In addition to its hydrophilic and gel-forming properties, HPMC has also been recognized for its mucoadhesive potential, which enables prolonged contact with mucosal surfaces [35,36,37]. Q. Zhang et al. have investigated the relationships between the physicochemical properties of tablets prepared from various commercial grades of hydroxypropyl methylcellulose (HPMC) and their in vitro mucoadhesive performance. They have shown the positive correlations between the viscosity of HPMC tablets and both the contact angle in different simulated body fluids and the in vitro mucoadhesive strength [38].

Several studies have demonstrated that combining different polymers can produce synergistic effects that enhance both mucoadhesive strength and drug release profiles. Because most commercial mucoadhesive formulations contain multiple excipients, we investigated binary mucoadhesive polymer systems at a 1:1 weight ratio. We hypothesized that these blends would enable the early identification of synergistic combinations with favorable surface free energy and hydration characteristics.

In this study, we focused on the surface properties of matrices containing HPMC with the addition of other widely used polymers, namely Carbopol 974P NF and Kollidon VA 64. Carbopol polymers are extensively used in pharmaceutical formulations, including controlled-release tablets, oral suspensions, and advanced drug delivery systems [39,40,41]. These polymers demonstrate significant mucoadhesive properties, particularly in acidic environments where they remain in their protonated state [42,43]. Carbopol 974P NF is a highly crosslinked polymer synthesized with allyl ethers of polyalcohols. Due to their three-dimensional crosslinked structure, carbomers do not dissolve in aqueous solutions but instead swell upon hydration, forming a highly viscous gel matrix [44,45]. Kollidon VA 64 is a copolymer of vinylpyrrolidone and vinyl acetate and is primarily used as a matrix-forming excipient in sustained-release drug formulations. It plays a crucial role in enhancing the solubility and bioavailability of poorly water-soluble active pharmaceutical ingredients (APIs) [46]. Its fine particle size distribution facilitates rapid dissolution and uniform dispersion, making it particularly advantageous for applications requiring precise drug delivery and improved formulation stability [47,48]. Specifically, its excellent solubility in water and other solvents makes it a versatile excipient for almost all dosage forms. Its wetting and binding powers are an advantage for many granulation technologies and tableting operations as they lead to compacts with higher tensile strengths [49]. Therefore, HPMC-based binary blends with Carbopol or Kollidon were selected to evaluate how polymer composition influences surface energetics, hydration, and mucoadhesion.

We hypothesized that blending HPMC, a cellulose-based polymer, with Carbopol or Kollidon would alter matrix hydration and surface energetics, thereby modulating mucoadhesion and structural stability in simulated salivary media, with potential implications for oral mucoadhesive matrices.

## 2. Materials and Methods

### 2.1. Materials

HPMC was obtained from Sigma-Aldrich (Merck, Poznań, Poland) and had an average molecular weight of approximately 86,000 Da. Kollidon VA 64 fine (hereafter referred to as ‘Kollidon’) was a generous gift of BASF (Ludwigshafen, Germany). Carbopol 974P NF was obtained from Lubrizol (Brussels, Belgium). The chemical structures of polymers are shown in Table 1 and their applications are presented in our previous papers [22,23]. The simulated saliva fluid was used in the study as the medium [50]. Simulated saliva fluid without mucin (SSF) and its modified version containing 0.1% mucin (SSF_muc_) were used to evaluate the effect of glycoprotein on the properties of the investigated polymer disks. Mucin stock solutions (Sigma Aldrich, Poznań, Poland), 2% and 4% (*w*/*v*), were prepared on the day of the measurement or one day before and were stored in a refrigerator at 5 °C. The mucin concentration of 0.1% (*w*/*v*), corresponding to 1 mg/mL, was selected to provide a glycoprotein-enriched saliva-mimicking medium close to reported mucin levels in resting human saliva, while maintaining adequate optical clarity and viscosity for contact-angle and SDi2 measurements. This medium was intended to reproduce mucin-related interfacial functionality rather than the full structure of the oral mucus layer. Therefore, separate 2% and 4% (*w*/*v*) mucin solutions were used for detachment-force experiments to represent a more concentrated mucin-rich adhesive interface during direct polymer–mucin contact.

Additional information regarding the composition of the measuring liquids is provided in the Supplementary Materials.

### 2.2. Preparation of Polymeric Disks

For characterization of the mucoadhesive polymers and their 1:1 (*w*/*w*) blends, disks with a diameter of 13 mm were prepared by direct compression of 200 mg powder at 2 MPa, 5 MPa or 8 MPa using a hydraulic press (2 Ton High Profile E-Z PressTM, International Crystal Laboratories, Garfield, NJ, USA) for 60 s.

Table 2 presents the compositions of the investigated formulations. The disks were stored at room temperature in a desiccator before use. The 1:1 (*w*/*w*) ratio was selected as a mechanistic screening composition because it provides equal mass contribution from HPMC and the second polymer and therefore enables direct comparison of the effects of Carbopol and Kollidon on hydration, surface energetics, and mucoadhesion. Other ratios were not included at this stage, as the objective was to identify polymer-specific mechanisms rather than to optimize a final dosage-form composition.

### 2.3. Measurement of Wettability

Advancing contact angle measurements on polymeric disks were performed using the sessile drop method with an optical tensiometer (Theta Lite, One Attension, KSV Nima, Espoo, Finland). All the measurements were conducted at 293 ± 0.1 K. A drop of liquid was automatically dispensed from the capillary and deposited onto the solid surface of the disk, and the drop profile was analyzed by fitting the experimental meridian to the theoretical drop shape based on the Young–Laplace equation. Images of the liquid drop were captured using a video-based contact angle tensiometer. Each measurement was repeated six times for each type of polymeric disk.

### 2.4. OWRK Method

The Owens–Wendt–Rabel–Kaelble (OWRK) method was applied to determine the surface free energy (SFE) of solid polymeric materials from contact angle measurements. This method separates the total surface free energy into dispersive and polar components. Contact angles were measured using two probe liquids of known surface tension—water and diiodomethane (Sigma-Aldrich, Poznań, Poland)—to evaluate the wettability of the investigated surfaces (see Table 3). The dispersive component (*γ*^d^) corresponds to non-specific, non-polar interactions, primarily van der Waals forces (including London dispersion forces), which are present in all materials. In contrast, the polar component (*γ*^p^) accounts for specific, orientation-dependent interactions, such as dipole–dipole interactions and hydrogen bonding, which arise in materials with permanent dipole moments. By employing both polar and non-polar test liquids, the OWRK method enables quantitative determination of each component’s contribution to the total surface free energy.

The method calculates the surface energy components of the solid using a mathematical model based on Young’s equation and the work of adhesion.

(1) $${\left(\gamma_{sv}^{d}\right)}^{0.5}=\frac{\gamma_{d}\left(cos\theta_{{CH}_{2}I_{2}}+1\right)-\sqrt{\frac{\gamma_{d}^{p}}{\gamma_{w}^{p}}}\cdot \gamma_{w}(cos\theta_{H_{2}O}+1)}{2\left(\sqrt{\gamma_{d}^{d}}-\sqrt{\gamma_{d}^{p}\frac{\gamma_{w}^{d}}{\gamma_{w}^{p}}}\right)}$$

(2) $${\left(\gamma_{sv}^{p}\right)}^{0.5}=\frac{\gamma_{w}\left(cos\theta_{H_{2}O}+1\right)-2\sqrt{\gamma_{sv}^{d}\gamma_{w}^{d}}}{2\sqrt{\gamma_{w}^{p}}}$$

where *γ_sv_*—the solid-vapor surface tension; *γ_w_*—the water surface tension; *γ_d_*—the diiodomethane surface tension and θ is the contact angle, which is established by a tangent to the liquid droplet and the solid surface in the area of intersection of the solid-liquid-vapor phases ($\theta_{{CH}_{2}I_{2}}$—the contact angle for diiodomethane, $\theta_{H_{2}O}$—the contact angle for water). The total surface free energy (*SFE*) of a solid equals:

(3) $$SFE=\gamma_{sv}^{d}-\gamma_{sv}^{p}$$

where $\gamma_{sv}^{d}$—dispersive part of the solid surface tension; $\gamma_{sv}^{p}$—polar part of the solid surface tension.

### 2.5. Measurements of Mucoadhesive Strength

The maximum detachment force was determined using a Texture Analyzer (Shimadzu AGS-1kNX), (Kyoto, Japan). The study involved polymeric disks (13 mm diameter, m = 200 mg) composed of either single polymers or polymer blends, which were affixed to a stainless steel probe (20 mm diameter). A mucin solution (2% or 4%) with a volume of 2 mL was applied to a Petri dish. The probe was lowered until the disk contacted the mucin solution and was held in place for 30 s to allow the formation of an adhesive bond between the polymer and mucin. Simultaneously, the disk was pressed against the solution surface with a constant force of 0.1 N. The probe with the attached disk was raised at a speed of 10 mm/min until the disk detached from the mucin solution. The maximum force required to break the adhesive bonds was recorded. The mucoadhesive strength was calculated based on the force–distance curve by the program TrapeziumX, version 1.5.2 (Shimadzu) (Kyoto, Japan).

### 2.6. Measurements of Swelling in 85% RH

The climate chamber (JeioTech, Korea TH-ICH-800) (Daejeon (Taejŏn), South Korea) works at the humidity and temperature of 85% and 25 °C, respectively. Disks were weighed individually to determine the initial mass. The samples were placed in hermetically sealed containers to prevent potential contamination. The vials were stored in the climate chamber under the specified temperature and humidity conditions. At predetermined time intervals (every 24 h over a 4-day period), tablets were retrieved, and their mass was measured using an analytical balance. For each type of disk 5–8 measurements were performed. The swelling index (SI) was calculated to assess water absorption capacity under controlled humidity conditions using the following equation:

(4) $$Swelling\;Index\left(SI\right)=\frac{\left(W_{t}-W_{0}\right)}{W_{0}}\cdot 100\%$$

where W_0_ is the initial weight of the dry disk, W_t_ is the weight of the swollen disk after time, and t = 96 h.

### 2.7. Surface Dissolution Imaging, SDi2

Surface dissolution imaging, SDi2 (Pion Inc, Forest Row, UK), was used to visualize changes in polymer disk morphology during exposure to simulated saliva containing mucin. A polymer disk was positioned vertically in a wire holder and put to the whole dosage flow cell filled with 1 mm glass beads to ensure laminar flow, exposing the face of the disk to the window. The test was performed in the closed loop configuration with 150 mL of mucin-containing SSF with mucin at 37 °C and a flow rate of 4 mL/min. Real-time imaging was performed at the visual wavelength of 520 nm for the target experiment time of 12 h (image size 2048 by 2048 pixels, frequency 1 image per second, every 50th frame saved). Based on the absorbance records, changes in disk dimensions were calculated by the SDi2 analysis software (v. 3.0.22), setting the tablet edge threshold value empirically at 200–250 mAu to mark the border between the disk and the medium, including the gel layer if present.

Adhesion testing and humidity-chamber water uptake were performed under controlled static conditions, whereas SDi2 experiments were conducted under dynamic flow using SSFmuc at 37 °C and a flow rate of 4 mL/min. Thus, the methodological design combined standardized static measurements for material comparison with dynamic imaging to evaluate structural stability during continuous exposure to a saliva-mimicking medium.

Data are presented as mean ± standard deviation where applicable. Contact-angle measurements were performed in six independent repetitions for each polymeric surface/liquid system (n = 6), water-uptake measurements in five to eight repetitions (n = 5–8), and mucoadhesive-force measurements in eight to ten repetitions (n = 8–10). Since the study was designed as a mechanistic preformulation screening and the applied techniques provide different types of analytical outputs, the results were interpreted using descriptive statistics, variability ranges, and convergence of trends across independent methods. Accordingly, the conclusions are based on reproducible patterns observed across contact-angle/SFE, water-uptake, mucoadhesive-force, and SDi2 analyses rather than on formal inferential comparisons between all datasets.

## 3. Results and Discussion

### 3.1. Preparation of Mucoadhesive Disks

**Figure 1:**
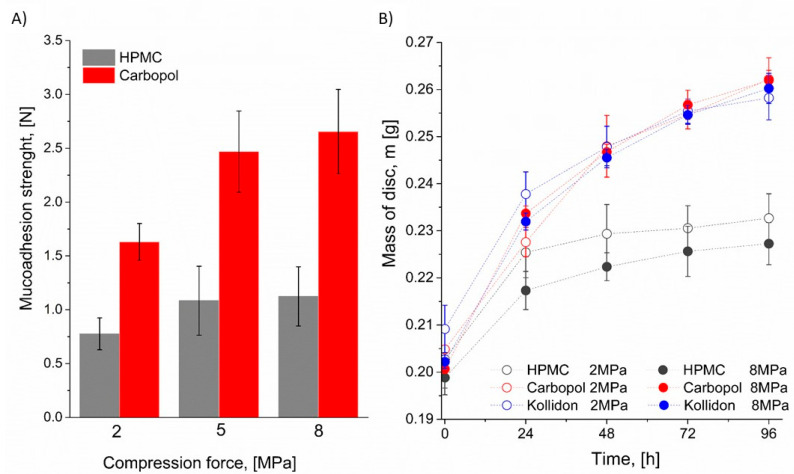
Impact of compression force on (**A**) mucoadhesion strength and (**B**) water absorption; n = 5–8.

Initial analyses were conducted to evaluate the effect of compression force during disk formation on water absorption and mucoadhesive strength. The disks were formed under 2, 5, and 8 MPa. For these systems we have studied the mucoadhesive strength and absorption of water in the climate chamber with parameters RH = 85% and T = 25 °C. The results are presented in Figure 1. A 2% (*w*/*v*) mucin solution was selected for the mucoadhesive strength studies. This choice can be justified by the fact that higher concentration closely mimics the physiological conditions of mucosal surfaces, particularly in the oral cavity, making the results more representative of in vivo environments. Lower concentrations may not form a sufficient mucosal layer, potentially leading to unstable or inaccurate adhesion measurements. Unfortunately, for the disk formed with Kollidon, we were unable to determine the maximum mucoadhesive force detachment (MDF) value, as all the formulations underwent excessive dissolution in the 2% mucin solution. However, the results obtained for HPMC and Carbopol clearly demonstrate that a higher compression force leads to an increased mucoadhesive strength. This effect was particularly pronounced for Carbopol, for which a 6 MPa increase in compression force resulted in an approximately 63% increase in mucoadhesive strength. For HPMC, the same increase in compression force led to an approximately 45% increase in mucoadhesive strength. Our findings are consistent with the results reported by Madhu S. Surapaneni et al. [51], who also demonstrated that, compared to HPMC, carbomer generates stronger adhesive forces upon hydration. In the literature, the difference in mucoadhesive force between carbomer and HPMC formulations is often attributed to the ability of carbomer to form hydrogen bonds with proton-accepting groups, a feature absent in HPMC due to its lack of proton-donating carboxyl groups [52,53,54].

Simultaneously, studies on water absorption were conducted (for detailed results and discussion, see Section 3.4). The prepared disks were placed in a climatic chamber for a period of four days, with mass measurements taken every 24 h to monitor water absorption. The results are presented in Figure 1B. The most significant increase in mass for all the systems was observed during the first 24 h, with slightly higher values recorded for the disks compressed at 2 MPa. Among the tested materials, the HPMC disks demonstrated the greatest stability and the lowest water uptake, with a mass increase of approximately 15% after four days. In contrast, the Carbopol 974P NF and Kollidon VA 64 disks exhibited very similar absorption profiles, reaching an average mass increase of about 30% over the same period. Beyond the initial 24 h, the influence of compression force on water absorption was no longer significant. The effect of compression and the associated changes in material capillarity were only evident during the first day. Notably, in the case of HPMC, no further mass increase was observed between 2 and 4 days, likely due to the formation of a thin gel layer on the tablet surface. This layer appears to act as a barrier, markedly reducing the diffusion of water molecules into the interior of the disk.

These results are partially consistent with those reported by Perioli [8,55], who investigated the influence of compression force on the behavior of mucoadhesive buccal tablets formulated with hydroxyethyl cellulose (HEC) and Carbopol 940. In the cited work, both mucoadhesive performance and drug release kinetics were influenced by compression force, although water penetration and polymer chain relaxation were not significantly affected. Specifically, higher compression forces resulted in reduced in vitro and in vivo drug release rates while enhancing in vivo mucoadhesion and hydration time. Interestingly, tablets compressed at the lowest force were associated with better overall performance and user comfort, whereas those prepared with the highest forces caused discomfort during application and required manual removal by study participants. In summary, these results suggest that while higher compression may initially enhance mucoadhesive strength and early hydration characteristics, excessive compaction can negatively affect long-term water uptake and patient acceptability. This underscores the importance of optimizing compression parameters to balance mucoadhesion, hydration, and comfort in buccal drug delivery systems.

Moreover, in our study the disks obtained at 2 MPa compression were easily ejected from the die cavity, indicating that the addition of a lubricant was not necessary within the parameters of this experimental setup. The resulting compacts exhibited smooth, glossy surfaces and showed no signs of capping or chipping, particularly around the edges, confirming their mechanical integrity and uniformity. The thickness of the tablets averaged 2 mm and was considered appropriate for sublingual application. Taking this into consideration, a compression force of 2 MPa was selected for further studies, as it provided a favorable balance between mucoadhesion, water absorption, and structural integrity.

### 3.2. Wetting Properties of Investigated Systems

**Figure 2:**
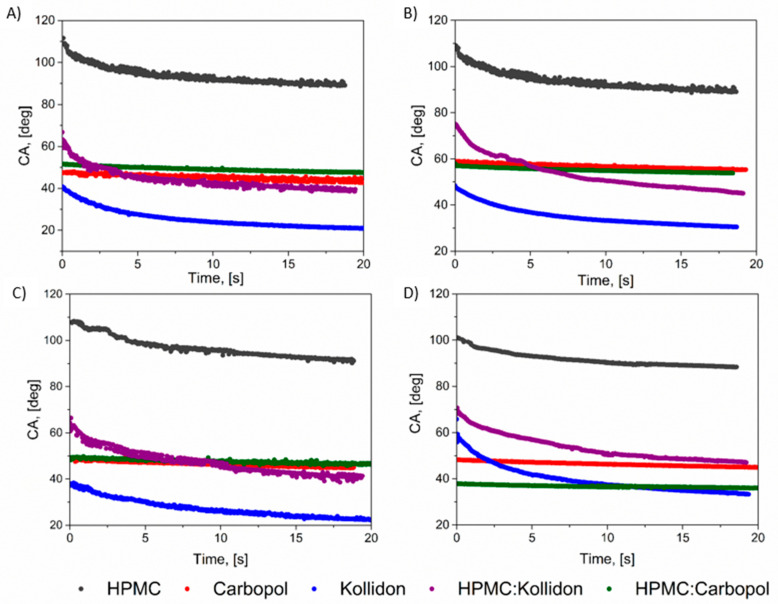
Contact angle curves for (**A**) water, (**B**) 2% *w*/*v* mucin solution, (**C**) simulated salivary fluid (SSF), and (**D**) SSF containing 0.1% mucin (SSF_muc_) measured on polymer disks and their equilibrium blends. The curves illustrate the wetting behavior over 20 s highlighting differences in surface hydrophilicity and the influence of mucin-containing fluids on the dynamics of contact angle.

Wettability is a crucial factor for successful mucoadhesion. Improved wettability promotes intimate contact between the dosage form and mucosal tissue, which can strengthen the mucoadhesive bond. A well-wetted surface may also enhance drug retention, support extended release, and improve bioavailability, thereby increasing the effectiveness of the mucoadhesive system. Figure 2 presents the results of the contact angle (CA) on time for the following fluids: water, 2% *w*/*v* mucin solution, saliva without mucin (SSF) and saliva with 0.1% mucin (SSF_muc_) [50]. The detailed chemical composition of the measurement liquids is provided in the Supplementary Material (Section S1).

For both the individual polymer disks and their blends, wetting behavior was similar when water or simulated salivary fluid (SSF) was used as the test medium. Among the tested materials, HPMC exhibited the most hydrophobic character, with an initial contact angle of approximately 110° that stabilized at around 90° after 20 s. In contrast, Carbopol showed a significantly lower initial contact angle of about 50°, which decreased only slightly during the 20-s measurement period, indicating moderate hydrophilicity. The most hydrophilic material was Kollidon, with an initial contact angle of approximately 40°, followed by a sharp decrease to around 20° over the 20-s wetting period, reflecting the most dynamic wetting behavior. The presence of 2% (*w*/*v*) mucin (Figure 2B) increased the initial contact angles by approximately 10° in the initial contact angles for Kollidon and Carbopol disks, compared with water or SSF. However, this modification (presence of mucin) did not substantially alter the overall dynamics of the wetting process. The observed trends in the contact angle curves for Carbopol and Kollidon are consistent with those reported in our previous studies [22,23]. The discrepancies in absolute contact angle values can be attributed to differences in the compression force applied during disk preparation. In our earlier work, a compaction force of 1.4 MPa was used, whereas in the current study, a higher force of 2 MPa was applied. The significance of compression force in contact angle measurements has been demonstrated in our previous study using Noveon disks [21].

Compared with our earlier studies, the present wetting results confirm the reproducibility of Carbopol behavior and emphasize the distinct behavior of HPMC. In our previous work [22], Carbopol 974P NF showed a water contact angle of 49.1°, which is very close to the value of 47.2 ± 2.3° obtained here despite the higher compaction pressure used in the present study. Kollidon showed a lower water contact angle in the current study (36.0 ± 1.8°) than previously reported for Kollidon VA 64 (40.9°) [22], which may reflect pressure-sensitive surface densification and exposure of polar groups during compaction. In contrast, HPMC showed a substantially higher water contact angle (103.4 ± 2.7°) than HEC reported in our previous saliva/vaginal-fluid study (20.7°) [22], demonstrating that cellulose derivatives cannot be treated as a homogeneous group with respect to surface wetting after compression. These reference values are also consistent with those reported for individual polymer components in our API-containing formulation study [23], where Carbopol 974P NF, HEC, and Kollidon VA 64 showed water contact angles of 49.1 ± 2.8°, 20.7 ± 1.4°, and 40.9 ± 0.6°, respectively.

Table 4 presents the estimated surface free energy (SFE) values using the Owens–Wendt–Rabel–Kaelble (OWRK) method for the surfaces of individual polymer disks and their blends. The values of surface free energy, dispersive (γ^d^) and polar (γ^p^) components were estimated according to Equations (1)–(3) described in Section 2.4. Interestingly, when comparing the polar and dispersive components with those reported in our previous study [23], we observe that the compaction force has no significant effect on the surface free energy of Carbopol disks. However, a noticeable difference is observed for Kollidon one. The observed increase in surface free energy (SFE) of Kollidon (from 61.3 mJ/m^2^ in our previous study (at 1.4 MPa compression) [23] to approximately 70 mJ/m^2^ in the current experiment (at 2.0 MPa)) can be attributed to several pressure-sensitive physicochemical mechanisms such as polymer network densification [56], Shuttleworth effect in polymers [57], and the effect of compact pressure on surface properties, which, in result, contributes to changes in adsorption behavior with liquids [58]. Higher pressure may lead to partial molecular alignment or reorientation at the surface, increasing exposure of polar carbonyl groups. This effect can probably enhance hydrogen bonding potential and increase the polar component of SFE. Moreover, a more compact surface may reduce microroughness and air entrapment during contact angle measurements, leading to more accurate (and often lower) contact angles, which correspond to higher calculated SFE. For the Kollidon, we observed that an increase in compact pressure causes a decrease in the water contact angle (WCA) of about 6 deg. in comparison to our previous data [23]. The surface free energy (SFE) of pure HPMC was found to be relatively low, with a remarkably high polar component accounting for approximately 95% of the total SFE. The incorporation of either Carbopol or Kollidon into the HPMC matrix led to a substantial increase in the dispersive component of the SFE. As a result, the binary polymer blends exhibited significantly lower polarity compared to the pure HPMC disks, indicating a pronounced shift in surface character due to polymer–polymer interactions.

A descriptive correlation analysis was additionally performed between the SFE components and the mean mucoadhesive-force values obtained for HPMC, Carbopol, HPMC:Carbopol, and HPMC:Kollidon. The results were included in the Supplementary Material (Tables S4 and S5). Kollidon was excluded from this comparison because its rapid disintegration prevented reliable detachment-force quantification. The relationship with mucoadhesive strength was stronger for the polar SFE component (Pearson r approximately 0.84) than for the dispersive component (r approximately 0.44) or total SFE (r approximately 0.48). Owing to the limited number of comparable systems, these correlations should be interpreted descriptively; nevertheless, they support the conclusion that specific polar interactions and functional-group availability are more relevant to mucoadhesion than total SFE alone.

**Figure 3:**
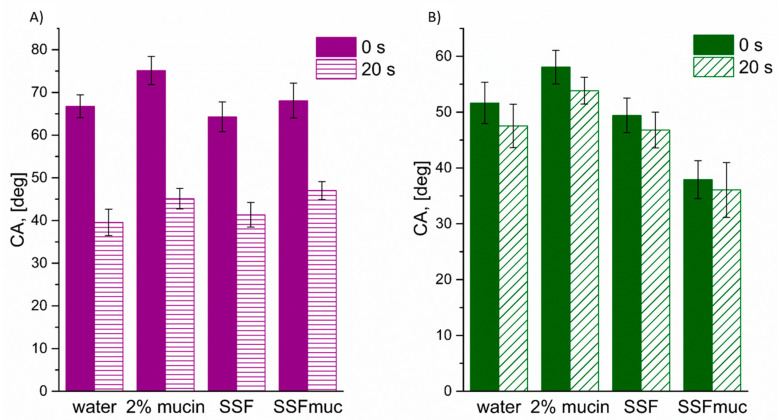
The contact angle for water, 2% mucin, SSF and SSFmuc on blend surface (**A**) HPMC:Kollidon and (**B**) HPMC:Carbopol at the beginning of the wetting process (t = 0 s) and after 20 s.

To facilitate a clearer comparison of the wettability of the binary blends, Figure 3 presents the contact angle values measured immediately upon contact and after a short interaction time for various testing liquids. For the HPMC:Carbopol system, the contact angle values measured at 0 and 20 s were very similar, independently of the type of testing liquid used (Figure 3B). In contrast, the HPMC:Kollidon blend exhibited noticeable wetting dynamics, characterized by a significant decrease in contact angle over the 20-second interval, typically by approximately 20° for all the considered wetting liquids (Figure 3A). Overall, at the initial stage of wetting, the HPMC:Carbopol blend exhibited superior wettability with all the tested liquids compared to the HPMC:Kollidon system. After 20 s, HPMC:Carbopol remained more effectively wetted by SSF_muc_, suggesting its enhanced affinity for mucin-containing environments and highlighting its potential suitability for mucoadhesive applications. This improved performance may be attributed to the strong hydrophilic and anionic nature of Carbopol, which is known to interact favorably with mucin glycoproteins through hydrogen bonding and electrostatic interactions [59]. Additionally, the high swelling capacity of Carbopol contributes to the formation of a hydrated gel layer that facilitates intimate contact with the mucosal surface and prolongs residence time [10]. In contrast, Kollidon is primarily governed by dispersive interactions and lacks the ionizable functional groups necessary for forming strong mucoadhesive bonding [46]. At the molecular level, the HPMC:Carbopol system combines the gel-forming continuity of HPMC with the high density of carboxylic groups in Carbopol. This combination may favor hydrogen-bonding and electrostatic contributions during polymer–mucin contact, while also limiting uncontrolled erosion through formation of a more cohesive hydrated layer. In contrast, Kollidon VA 64, although rapidly wetted, is non-ionic and lacks functional groups capable of strong ionic interaction with mucin; therefore, rapid hydration may translate into matrix weakening rather than durable mucoadhesion.

Therefore, its combination with HPMC may not yield the same degree of mucin affinity as observed for HPMC:Carbopol.

### 3.3. Mucoadhesive Properties of Polymer Disks

**Figure 4:**
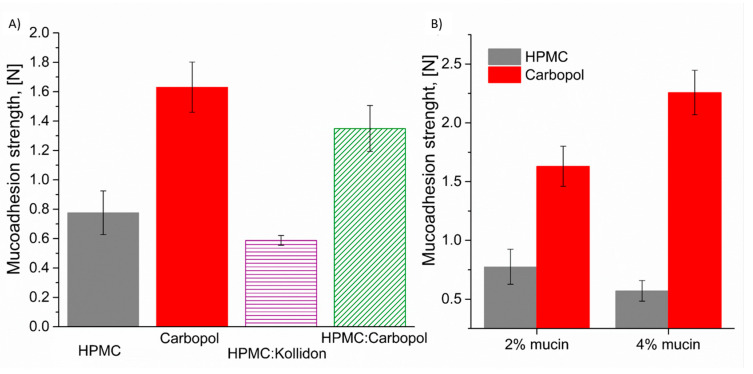
(**A**) Mucoadhesive force values of individual polymers and their binary blends measured in the presence of a 2% (*w*/*v*) mucin solution. Measurements were conducted using a texture analyzer with an applied contact force of 0.1 N for 30 s and a probe detachment speed of 10 mm/min. (**B**) The effect of mucin concentration (2% and 4% *w*/*v*) on the mucoadhesive force of polymer surfaces; n = 8–10.

Evaluation of mucoadhesive strength plays a crucial role in understanding the performance of polymers intended for mucosal drug delivery. This parameter reflects the ability of a formulation to maintain intimate contact with mucosal surfaces, and may therefore enhance drug absorption and prolong residence time at the site of administration. In our study, the comparison of mucoadhesive strength between individual polymers and their binary blends offered valuable insights into the influence of polymer composition on adhesion. Figure 4B presents the mucoadhesive force values of the individual polymer, highlighting the effect of mucin concentration in the testing medium. Mucoadhesion testing under the specified conditions could not be reliably performed for Kollidon, as the results were subject to significant error due to the rapid disintegration of the polymer matrix during the 30 s contact time of the Kollidon disk with 2% and 4% (*w*/*v*) mucin solution. This instability compromised the consistency of the measurements and precluded the accurate quantification of the detachment mucoadhesive force.

Figure 4B illustrates the effect of mucin concentration on mucoadhesive force. For the HPMC disk, no significant differences in mucoadhesive strength were observed after a twofold increase in mucin concentration. The average mucoadhesive force for the HPMC disks compressed at 2 MPa was estimated to be 0.5–0.75 N. In contrast, Carbopol showed a pronounced mucin concentration effect: a twofold increase in mucin concentration resulted in an approximately 40% enhancement in mucoadhesive strength. This finding suggests that Carbopol has a greater capacity for interfacial interactions with mucin, likely due to its high density of carboxylic groups capable of forming hydrogen bonds and electrostatic interactions. The inherent properties of individual mucoadhesive polymers are subsequently reflected in the analysis of the impact of blend composition on mucoadhesive strength (Figure 4A). It is clearly evident that the HPMC:Carbopol blend exhibits superior mucoadhesive performance compared to only HPMC. Moreover, it exhibits superior mucoadhesive properties compared to the HPMC:Kollidon blend. The mucoadhesive properties observed for the HPMC:Carbopol blend in the 2% mucin solution can be explained by the complementary interaction mechanisms between these polymers and mucin. Carbopol is an anionic polymer characterized by a high density of carboxylic acid groups, which facilitates strong hydrogen bonding and electrostatic interactions with the negatively charged mucin glycoproteins [10]. These interactions enhance the adhesion strength significantly compared to systems lacking ionizable groups. On the other hand, Kollidon is a non-ionic polymer with predominantly dispersive interactions and lacks ionizable functional groups necessary for strong ionic bonding with mucin. While HPMC contributes to mucoadhesion primarily through hydrogen bonding and hydrophilicity, the inclusion of Carbopol introduces additional electrostatic interactions that synergistically improve overall mucoadhesive strength [58]. This explains why the HPMC:Carbopol blend exhibits markedly better mucoadhesive performance than the HPMC:Kollidon one under the tested conditions. These observations are consistent with literature reports emphasizing the role of polymer charge and functional groups in governing mucoadhesive behavior [60,61].

It should be noted that the proposed contribution of hydrogen bonding and electrostatic interactions is inferred from the chemical functionality of the polymers, wettability behavior, surface free energy components, hydration characteristics, and mucoadhesive-force measurements. Although these complementary results consistently support the involvement of specific polymer–mucin and polymer–polymer interactions, direct spectroscopic confirmation was not included in the present experimental design. Therefore, future ATR-FTIR studies of HPMC, Carbopol, Kollidon, and their binary blends would be valuable to verify possible band shifts or broadening in the O–H, C=O, and C–O/C–O–C regions associated with hydrogen-bond formation.

Contact-angle/SFE analysis, mucoadhesive-force measurements, water uptake, and SDi2 imaging were used as complementary methods describing successive stages of hydration-mediated mucoadhesion: initial wetting, hydration capacity, polymer–mucin mechanical interaction, and matrix stability under flow.

### 3.4. Swelling and Stability of Polymeric Disks

Water sorption capacity is a critical factor influencing the functional performance of mucoadhesive polymer-based systems. Hydration initiates polymer swelling, which in turn enhances chain mobility and facilitates interpenetration with mucosal mucus; the ability of a material to absorb and retain moisture directly affects its mucoadhesive strength [59,61]. However, uncontrolled or excessive water uptake may also compromise the mechanical integrity of the dosage form, leading to premature erosion, changes in adhesion time, or instability during storage. To gain insight into the hygroscopic behavior of the tested materials, additional studies were performed under controlled conditions in a climate chamber at 85% relative humidity (RH), simulating elevated-moisture environments. These experiments allowed us to compare the water uptake profiles of individual polymers and their binary systems, and to assess the potential impact of moisture on the structural stability and mucoadhesive performance of the systems. The findings provide a valuable complement to wettability and mucoadhesion data, contributing to a comprehensive understanding of the behavior of these materials under physiological and storage-relevant conditions. Table 5 presents the swelling index values for all the polymers and blends considered in this study. The swelling index was calculated according to Equation (1) in Section 2.6.

The obtained swelling index values clearly indicate that HPMC absorbs approximately half the amount of water compared to Kollidon and Carbopol matrices. The disks prepared solely from Kollidon or Carbopol exhibited comparable water uptake values, ranging from 28 to 30%, when compacted at 8 MPa. Slight differences were observed for the disks compressed at 2 MPa, with Carbopol demonstrating marginally higher swelling under these conditions. The distinct behavior of the HPMC disk is likely attributable to the formation of a surface gel layer upon exposure to a highly humid environment.

Disks prepared solely from Kollidon or Carbopol exhibited comparable water uptake when compacted at 8 MPa. To address swelling kinetics more quantitatively, the 96 h swelling index values were treated as mass-based hydration descriptors rather than only as qualitative observations. At 2 MPa, the swelling index was 14.8 ± 0.5% for HPMC, 28.0 ± 0.4% for Carbopol, 23.5 ± 0.5% for Kollidon, 25.7 ± 0.3% for HPMC:Kollidon, and 29.4 ± 0.3% for HPMC:Carbopol. Thus, incorporation of Carbopol into the HPMC matrix approximately doubled the final water uptake compared with HPMC alone, whereas HPMC remained the least hydrated and most dimensionally stable material under high-humidity conditions.

Figure 1B (or for better clarity, Figure S3 in Supplementary Material) presents the increase in mass of the disk measured at 24-hour intervals. It has been clearly shown that water absorption by the HPMC matrix occurs predominantly within the first day of measurement, after which its mass stabilizes. The Supplementary Materials provide photographs illustrating the appearance of the studied disks, along with key preliminary observations (Figure S2). It could be assumed that a gel layer appears to act as both a physical and diffusional barrier, restricting the penetration of water molecules into the core of the polymeric matrix. As water initially infiltrates the outermost regions, HPMC chains begin to hydrate, swell, and uncoil, resulting in the rapid formation of a viscous gel layer [62,63]. Although highly hydrated, this gel exhibits lower porosity and reduced diffusivity compared to the dry core, significantly slowing the rate of further water penetration. Consequently, the inner regions of the matrix remain relatively less hydrated over time, leading to a limited extent of overall swelling. This phenomenon is consistent with previously reported diffusion-controlled hydration mechanisms observed in hydrophilic matrix systems [64,65], where early gel layer formation governs water uptake kinetics and matrix expansion. The results indicate that compaction pressure did not significantly affect the water absorption of individual polymer disks for the 24 h observation intervals.

**Figure 5:**
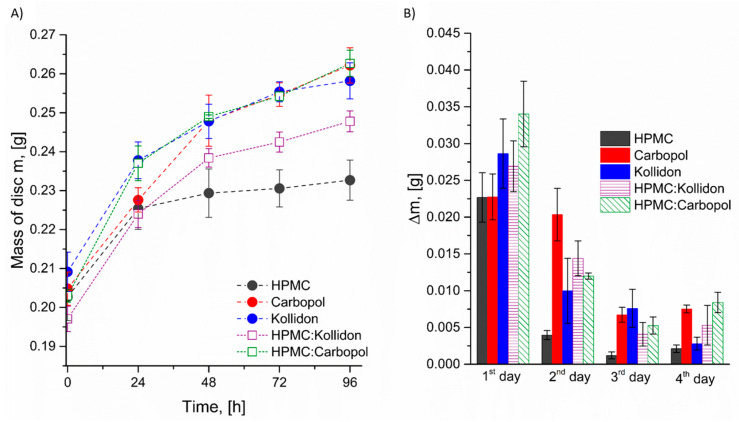
Water uptake profiles of individual polymers and their binary blends over a 4-day period: (**A**) changes in disk mass over time; (**B**) daily mass increase (Δm) during consecutive days. Measurements were conducted at RH = 85% and T = 25 °C using disks compressed at 2 MPa.

The addition of either Kollidon or Carbopol to HPMC was found to enhance water absorption within the polymer matrix (Figure 5). As expected, the most pronounced increase in mass was observed during the first 24 h for the investigated blends (Figure 5B). This enhanced water uptake can be attributed to the physicochemical characteristics of Kollidon and Carbopol. As previously demonstrated, these materials exhibit significantly higher surface energy compared to HPMC (Table 2), which indicates their greater hydrophilicity. Kollidon is a highly hydrophilic polymer with excellent water-binding capacity, which facilitates faster and more extensive water penetration into the matrix. Figure S2 (Supplementary Material) presents the appearance of the disks after removal from the climate chambers. In the case of Kollidon, pronounced deformation of the disks was observed, including strong adhesion to the vial walls. Ultimately, under the specified conditions, the Kollidon matrices completely dissolved after 96 h. Carbopol, on the other hand, is a crosslinked polyacrylic acid that swells significantly in aqueous environments due to its high density of ionizable carboxyl groups. When incorporated into the HPMC matrix, both polymers disrupt the dense gel layer typically formed by HPMC alone, which probably improves water diffusion and overall matrix hydration.

To further support the quantitative interpretation of swelling behavior, time-dependent SI values calculated from mass changes were fitted to an empirical power-law model, ${SI}_{t}=kt^{n}$; the fitted parameters and coefficients of determination are provided in the Supplementary Material (see Table S2 and Figure S4). The empirical power-law model provided a good descriptive fit for all the investigated swelling profiles, with R^2^ values of 0.958–0.993 for the log-linear regression. HPMC showed the lowest exponent (n = 0.194), which is consistent with rapid early hydration followed by a plateau-like trend. This agrees with the interpretation that a surface gel layer may limit further water penetration into the HPMC matrix. Carbopol 974P NF showed the highest exponent (n = 0.664), indicating the strongest time-dependent increase in swelling index over the investigated period. The HPMC:Carbopol blend showed a lower exponent (n = 0.369) than Carbopol alone, suggesting that the presence of HPMC moderated the swelling rate while still allowing substantial hydration. HPMC:Kollidon showed an intermediate exponent (n = 0.452), indicating pronounced time-dependent hydration; however, this should be interpreted together with the SDi2 observations showing reduced structural stability and erosion of Kollidon-containing matrices.

Overall, the fitted parameters support the conclusion that water uptake alone does not determine functional mucoadhesive performance. The most favorable behavior is associated with a balance between hydration, polymer–mucin interaction capacity, and matrix stability.

**Figure 6:**
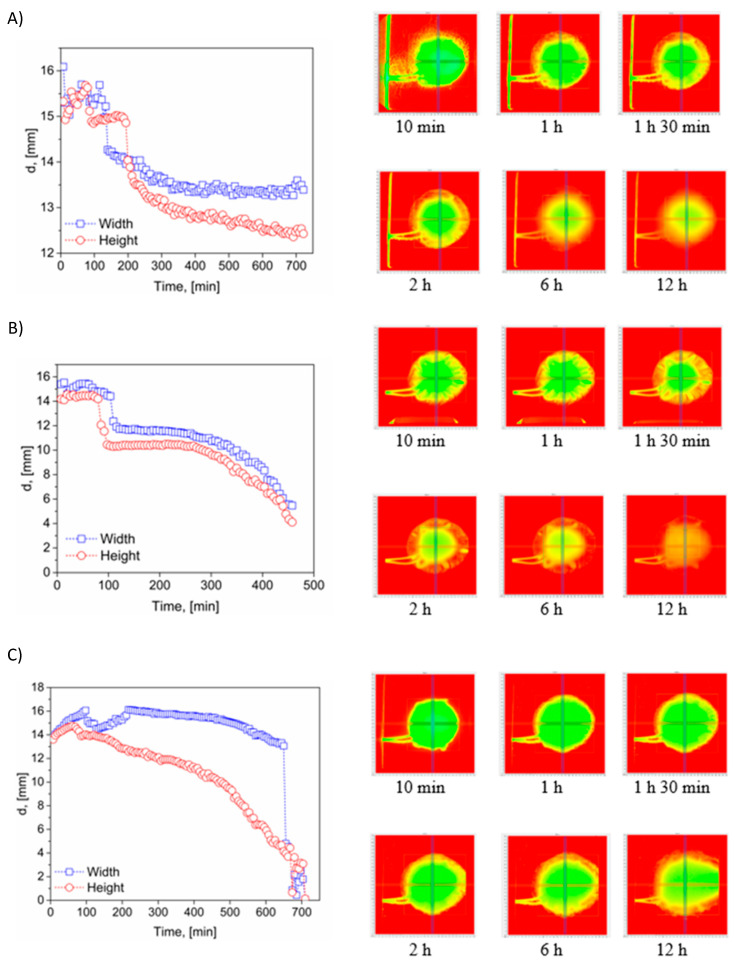
SDi2 images of experiments conducted at an absorbance of 520 nm. Red lines on photos show where the tracking box is measuring the “gel layer” at the selected zones: (**A**) HPMC, (**B**) HPMC:Kollidon and (**C**) HPMC:Carbopol. The colored line indicates the analyzed profile, the box marks the selected region of interest, and the colors represent the relative intensity distribution in the recorded images.

The hydration and swelling characteristics of the tested polymeric systems were further investigated using SDi2, PION, which enables real-time, high-resolution visualization of dimensional and morphological changes upon exposure to aqueous media. This technique facilitates dynamic monitoring of tablet surface geometry, yielding valuable insights into swelling kinetics, erosion patterns, and the structural integrity of dosage forms [66]. By analyzing the geometric transformations of HPMC and its binary blends with Carbopol or Kollidon, the influence of polymer composition on hydration-driven behavior was elucidated—an aspect directly linked to mucoadhesive performance and drug release dynamics. The integration of SDi2 imaging with mucoadhesive force measurements and water uptake studies provides a comprehensive assessment of the physicochemical properties of mucoadhesive formulations under conditions simulating the oral cavity. This combined approach enhances predictive understanding of formulation performance, particularly during the early-stage screening of promising polymer combinations for mucosal drug delivery applications. The obtained results were presented in Figure 6A–C for HPMC disks and blends: HPMC:Carbopol and HPMC:Kollidon in SSF_muc_ fluid. The Supplementary Material provides additional images of the disks captured at shorter time intervals to illustrate the progression of structural changes (Table S3). Height and width profiles obtained from disks stored in the flow-through chamber over a 12-hour period revealed that the most stable formulation was the HPMC-only disk, which developed a robust gel layer over time.

Throughout the 12-hour observation period, the dimensions of the HPMC disk changed proportionally by approximately 18% in both height and width of the disk. Incorporation of Kollidon clearly accelerated the disintegration process, with the HPMC:Kollidon disk being almost completely dissolved in the SSF_muc_ medium after approximately 6 h (Figure 6B). A particularly rapid decrease in geometric dimensions was observed after 4 h 30 min of exposure. The Kollidon disk alone exhibited rapid disintegration, accompanied by a significant decrease in density. As a result, after approximately 20 min, the disk could no longer be held in place within the measurement cell holder. Due to buoyancy forces, it was displaced and eventually pushed out of the optical window of the measurement chamber (see Supplementary Materials, Table S3).

Quantitative SDi2 image analysis was based on changes in disk height and width identified using an empirical edge threshold of 200–250 mAu. HPMC showed an approximately 18% proportional dimensional change over 12 h, corresponding to an average dimensional change of about 1.5% per hour. In contrast, HPMC:Kollidon underwent accelerated erosion and was nearly completely dissolved after approximately 6 h, whereas neat Kollidon lost positional stability in the optical window after approximately 20 min. These metrics confirm that rapid wetting does not necessarily indicate mechanical robustness during prolonged hydration.

In both the HPMC and HPMC:Kollidon systems, the hydration process resulted in noticeable asymmetric deformation of the disk structure, likely due to uneven swelling and directional erosion. In contrast, the incorporation of Carbopol led to the formation of a gel-like matrix with a slower dissolution rate compared to the HPMC:Kollidon formulation. In this case, a continuous reduction in the geometric dimensions of the disk was observed (Figure 6C), with the changes being more pronounced in the vertical (height) direction than in the horizontal (width) direction. After approximately 6 h, a sharp decrease in disk width was observed, indicating intensified gel formation and directional dissolution along the radial axis. The corresponding image of the disk clearly reveals axial deformation, reflecting the progressive and asymmetric structural disintegration of the matrix over time.

The asymmetric deformation observed in Kollidon-containing systems is attributed to non-uniform liquid penetration, local erosion, and directional weakening of the hydrated matrix. SDi2 is advantageous because it captures these processes in real time under flow, but it remains an optical, threshold-dependent technique and does not directly identify molecular interactions. Therefore, the SDi2 data should be interpreted together with contact-angle, water-uptake, and mucoadhesive-force measurements.

## 4. Conclusions

A comprehensive evaluation of HPMC, Carbopol, Kollidon, and their binary blends provided valuable insights into their mucoadhesive properties, hydration behavior, and structural stability under conditions simulating the oral cavity. Among the tested formulations, the HPMC:Carbopol system exhibited the most favorable mucoadhesive performance, particularly in the presence of mucin, probably due to complementary hydrogen bonding and electrostatic interactions mechanisms inferred from polymer structure and performance data. In contrast, the HPMC:Kollidon system showed weaker mucoadhesion and more rapid disintegration, consistent with the non-ionic character and predominantly dispersive surface properties of Kollidon. Surface energy analysis and contact angle measurements confirmed the critical influence of polymer composition on wettability and mucoadhesive potential. Water uptake studies combined with real-time imaging (SDi2) demonstrated that HPMC alone formed the most structurally stable gel over time, whereas the incorporation of Carbopol improved the structural integrity of the matrix, minimizing asymmetric deformation. In contrast, the systems containing Kollidon exhibited early onset of erosion and axial disintegration. These findings highlight the importance of polymer selection and compatibility in the design of stable mucoadhesive oral formulations. Importantly, the convergent trends obtained from contact-angle/SFE analysis, mucoadhesive-force measurements, water uptake, and SDi2 imaging indicate that HPMC:Carbopol provides the most favorable balance between wettability, hydration, mucoadhesive strength, and structural stability, whereas HPMC:Kollidon undergoes rapid wetting but shows weaker adhesion and lower resistance to erosion. Among the evaluated systems, HPMC:Carbopol blend emerged as a promising candidate for further development in mucoadhesive drug delivery applications. The results of our study confirm that a thorough physicochemical analysis is absolutely essential for the rational and effective selection of polymers for pharmaceutical formulations. It has been proven that understanding the relationship between the molecular structure of individual mucoadhesive polymers, their binary combinations, and resulting surface properties is essential for the rational design of effective mucosal drug delivery systems. Therefore, this study provides a mechanistic framework for predicting mucoadhesive performance based on physicochemical characteristics such as surface free energy and hydration behavior, thereby offering a valuable tool for the early-stage optimization of buccal formulations.

Improved mucoadhesion is expected to increase residence time at the oral mucosa and reduce premature removal by saliva, which may enhance local exposure and, depending on the active substance, bioavailability. From a mechanistic perspective, prolonged polymer–mucin contact may also support the formation of a hydrated interfacial gel layer, maintaining a localized concentration gradient at the epithelial surface and extending the time window available for drug diffusion or local pharmacological action. This translational implication should be verified in future drug-loaded and ex vivo or in vivo studies.

## Figures and Tables

**Table 1:** Representative chemical structures (repeating units) of HPMC, Carbopol 974P NF, and Kollidon VA 64 fine.

Polymer	Chemical Structure
HPMC (hydroxypropylmethylcellulose)	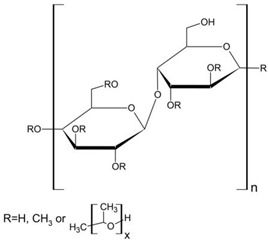 MW = 1 × 10^4^–1.5 × 10^5^
Carbopol 974P NF (corss-linked poly(acrylic acid))	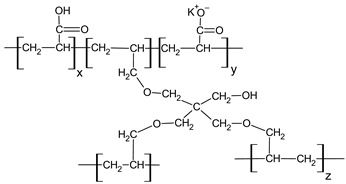 MW = 7 × 10^5^–4 × 10^9^
Kollidon VA 64 (vinylpyrrolidone-vinylacetate copolymer)	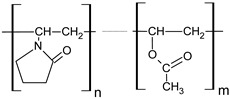 The ratio of n/m = 1.2; n, m—number of monomers in polymer chain MW = 4.5 × 10^4^–7.0 × 10^4^

**Table 2:** Investigated formulations: single mucoadhesive polymers and polymer blends.

Formulation	Polymers
Mucoadhesive polymers	HPMC Carbopol 974P NF Kollidon VA 64
Mucoadhesive blends (1:1, *w*/*w*)	HPMC:Carbopol 974P NF HPMC:Kollidon VA 64

**Table 3:** Surface tension of tested liquids: *γ*^p^—polar part of surface tension; *γ*^d^—dispersive part of surface tension.

Liquids	*γ* [mN/m]	*γ*^p^ [mN/m]	*γ*^d^ [mN/m]
Water (w)	72.8	51.0	21.8
Diiodomethane (d)	50.8	0	50.8

**Table 4:** Surface free energy (SFE) of polymers and their blends estimated using the OWRK method; *γ*^p^—polar and *γ*^d^—dispersive component of SFE; $\theta_{H_{2}O}$ water contact angle; $\theta_{{CH}_{2}I_{2}}$—diiodomethane contact angle; polarity—the percentage share of polar component in surface energy.

Mucoadhesive Polymer	$\theta_{H_{2}O}$ [deg]	$\theta_{{CH}_{2}I_{2}}$ [deg]	SFE [mJ/m^2^]	*γ*^p^ [mJ/m^2^]	*γ*^d^ [mJ/m^2^]	Polarity [%]
Kollidon	36.0 ± 1.8	15.7 ± 2.3	70.5	21.6	48.9	30.6
HPMC	103.4 ± 2.7	139.5 ± 3.1	12.0	11.3	0.7	94.2
Carbopol	47.2 ± 2.3	20.8 ± 2.9	64.1	16.4	47.7	25.6
HPMC:Carbopol	51.3 ± 2.8	13.4 ± 3.5	63.0	13.6	49.4	22.0
HPMC:Kollidon	53.9 ± 3.4	19.6 ± 2.8	60.7	12.8	47.9	21.0

**Table 5:** Swelling index values for polymer disks stored in a climate chamber for 96 h at RH = 85%, T = 25 °C.

Polymer/Blend	Compression Force [MPa]	Swelling Index [%]
HPMC	2	14.8 ± 0.5
Carbopol	2	28.0 ± 0.4
Kollidon	2	23.5 ± 0.5
HPMC:Kollidon	2	25.7 ± 0.3
HPMC:Carbopol	2	29.4 ± 0.3
HPMC	8	14.3 ± 0.4
Carbopol	8	30.5 ± 0.2
Kollidon	8	28.7 ± 0.3

## Data Availability

The original contributions presented in this study are included in the article/Supplementary Material. Further inquiries can be directed to the corresponding authors.

## Supplementary Materials

The following supporting information can be downloaded at https://www.mdpi.com/article/10.3390/polym18101227/s1, Figure S1: Measurement of the polymer discs mass and their blends using an analytical balance; Figure S2: Images of polymer and polymer blend discs, compressed at 2 MPa, after 24 and 96 hours of storage in a climate chamber (RH = 85%, T = 25 °C); Figure S3: Mass gain of polymer discs compressed at 2 MPa and 8 MPa, during storage under controlled conditions (RH = 85%, T = 25 °C); Figure S4: Points show calculated SI values based on mass changes; curves show the fitted empirical model: SI_t_ = kt^n^; Table S1: Input masses and calculated swelling index values; Table S2: Power-law model parameters for swelling profiles; Table S3: SDI2 images for polymers and their blends (1:1, *w*/*w*); Table S4: Input data used for the descriptive Pearson correlation analysis; Table S5: Summary of Pearson correlations.
