# Supplementary material for: Cellulose-Based Polymer Blends for Oral Mucoadhesion: Impact of Hydration and Surface Interactions
Source: Polymers (Basel). 2026 May 17;18(10):1227. doi: 10.3390/polym18101227 (PMC13210954; doi:10.3390/polym18101227)
Supplement: Supplementary file 1 [file polymers-18-01227-s001.zip › polymers-4310581-supplementary.pdf]

SUPPLEMENTARY MATERIALS

1. Composition of measurement liquids

Four different media were used to evaluate the wettability, mucoadhesive behavior, and hydration profiles of polymeric discs:

1. **Distilled water (H_2O)** – served as a neutral reference fluid. Surface tension at 21ºC=72.3 mN/m.
2. **2% or 4% (w/v) mucin solution** – prepared by dissolving porcine gastric mucin (Type III) in distilled water under gentle stirring at room temperature.
3. **Simulated Saliva Fluid (SSF)** :
   - 8.0 g/L NaCl
   - 0.19 g/L KCl
   - 2.38 g/L KH_2PO_4
   - 0.17 g/L $CaCl_2·2H_2O$
   - 0.2 g/L $MgCl_2·6H_2O$
   - Adjusted to pH 6.8 with NaOH.

   Surface tension at 21ºC=72.0 mN/m
4. **SSF_{muc}** – SSF supplemented with 0.1% (w/v) mucin.

All solutions were freshly prepared and equilibrated to room temperature before use.

2. Sorption and swelling measurement

The polymer discs were weighed in closed Eppendorf vials immediately after removal from the climate chamber to minimize exposure to ambient humidity. Following each measurement, the vials were promptly returned to the chamber and the caps were removed to restore equilibrium. The weighing process for a single vial did not exceed 2 minutes, ensuring that environmental fluctuations had negligible impact on the results.

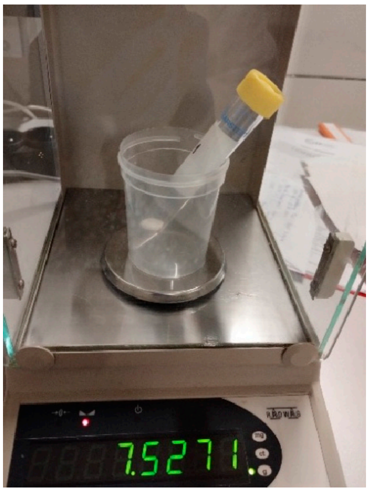

Figure S1 Measurement of the polymer discs mass and their blends using an analytical balance.

Observations:

- **HPMC:** No visible geometric changes of the discs.
- **Kollidon VA 64 (fine):** Clearly visible changes in disc geometry; discs become sticky and adhere to the vials after 24 hours; after 4 days, they are almost completely dissolved.
- **Carbopol 974P NF:** After 24 hours, slight changes in disc geometry are visible; the discs become sticky.
- **HPMC:Kollidon VA 64 (1:1):** After 96 hours, slight changes in disc geometry are observed; discs are slightly deformed and become sticky.
- **HPMC:Carbopol 794P NF (1:1):** Discs become stickier without any noticeable changes in geometry.

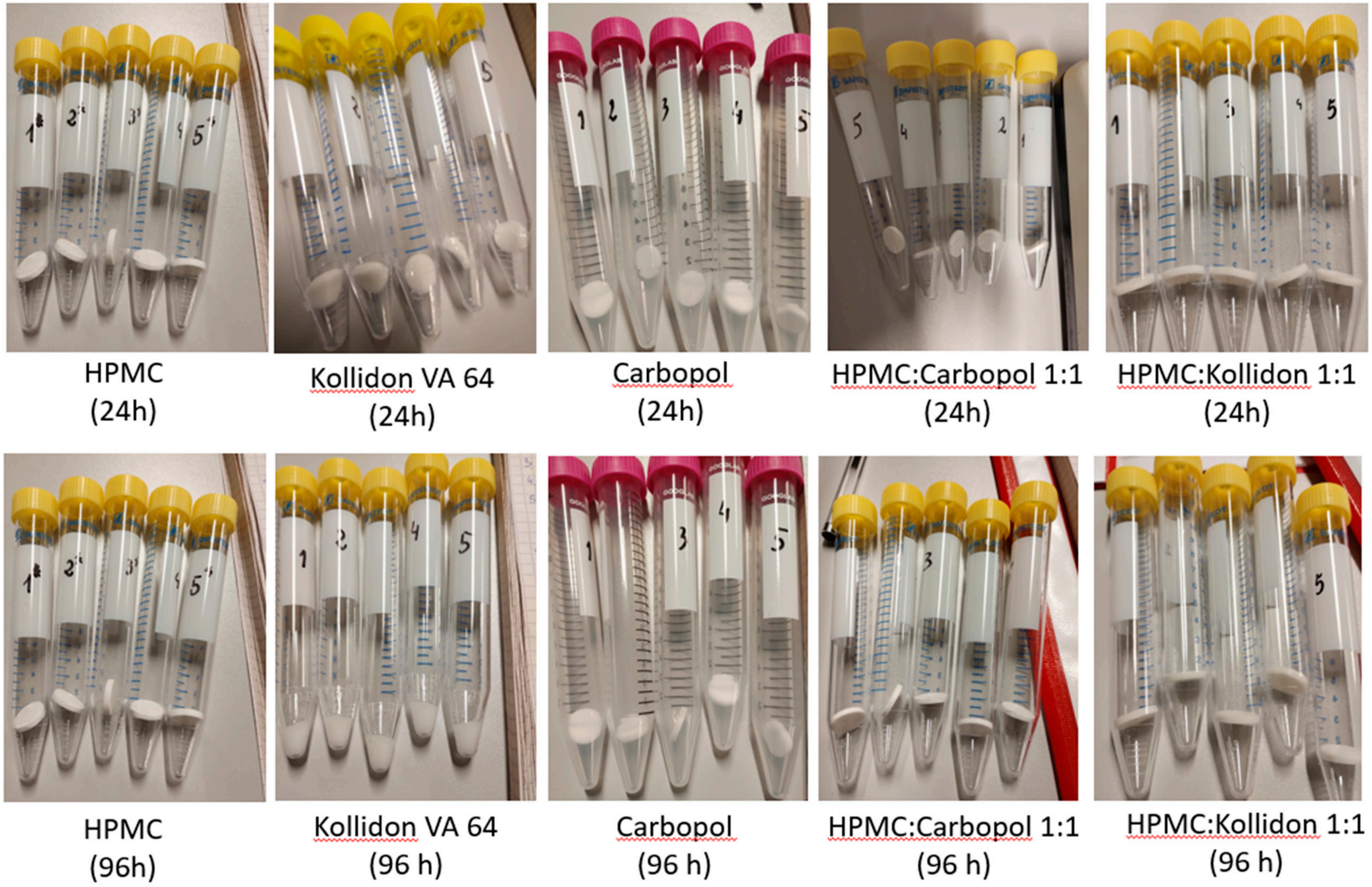

Figure S2 Images of polymer and polymer blend discs, compressed at 2 MPa, after 24 and 96 hours of storage in a climate chamber (RH = 85%, T = 25ºC).

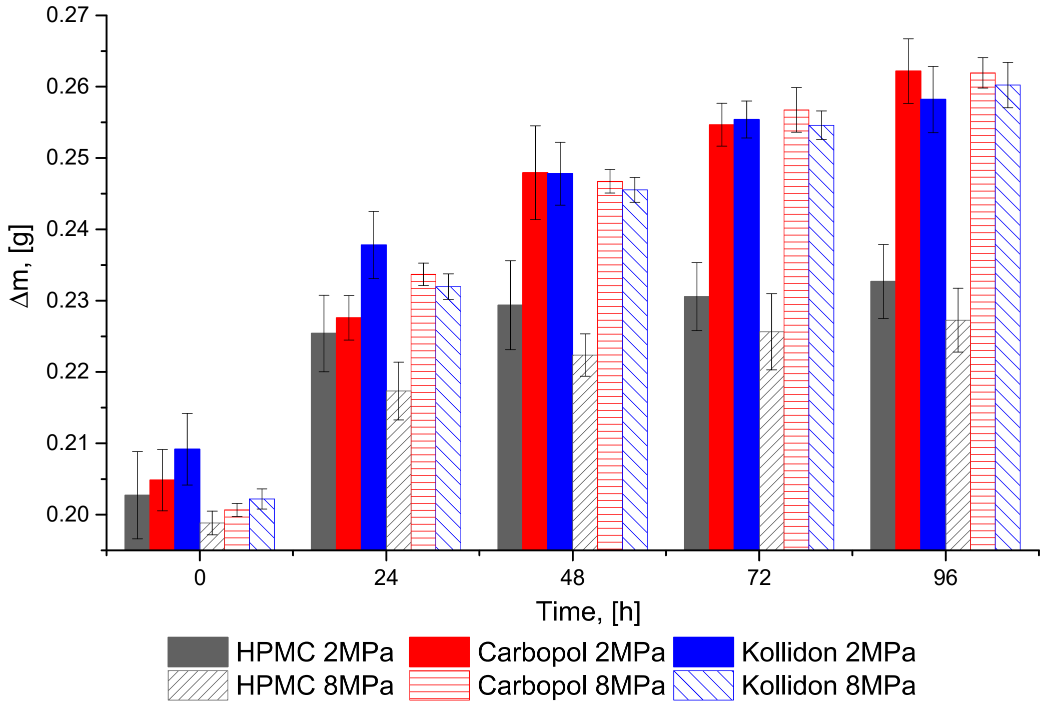

Figure S3. Mass gain of polymer discs compressed at 2 MPa and 8 MPa, during storage under controlled conditions (RH = 85%, T = 25°C).

3. Empirical Modeling of Swelling Kinetics Based on Time-Dependent Mass Changes

To provide quantitative support for the swelling observations, time-dependent mass changes were converted into swelling index values and fitted to an empirical power-law model, $SI_t = kt^n$. The fits showed good descriptive agreement with the calculated SI profiles (R^2 = 0.958-0.993, log-linear scale). The low exponent obtained for HPMC indicated rapid early hydration followed by limited further water uptake, whereas higher exponents for Carbopol and Kollidon-containing systems reflected more time-dependent water uptake. These results support the interpretation that matrix performance is governed not only by the magnitude of water uptake but also by the ability of the hydrated system to maintain structural integrity.

Calculation of swelling index

For each formulation, the mass measured at time 0 h was treated as the initial dry mass of the disc (W_0). The swelling index at each subsequent time point was calculated according to Eq. (S1).

$$\text{Swelling Index } (SI_t) = \frac{(W_t - W_0)}{W_0} \cdot 100\% \quad (S1)$$

where SI_t is the swelling index at time t, W_0 is the initial mass of the disc, and W_t is the mass of the disc after a given hydration time. The initial point (t = 0 h) was used only to define W_0 and was not included in the model fitting.

Empirical swelling model

The calculated SI values were fitted to the empirical power-law model shown in Eq. (S2).

$$SI_t = kt^n \quad (S2)$$

The model was fitted in its linearized logarithmic form according to Eq. (S3).

$$\ln(SI_t) = \ln(k) + n \cdot \ln(t) \quad (S3)$$

Table S1. Input masses and calculated swelling index values.

Formulation	Time (h)	Mass (g)	SI (%)
Carbopol 974P NF	0	0.20484	0.00
Carbopol 974P NF	24	0.22760	11.11
Carbopol 974P NF	48	0.24794	21.04
Carbopol 974P NF	72	0.25466	24.32
Carbopol 974P NF	96	0.26218	27.99
HPMC	0	0.20272	0.00
HPMC	24	0.22540	11.19
HPMC	48	0.22936	13.14
HPMC	72	0.23056	13.73
HPMC	96	0.23268	14.78
Kollidon VA 64	0	0.20916	0.00
Kollidon VA 64	24	0.23780	13.69
Kollidon VA 64	48	0.24780	18.47
Kollidon VA 64	72	0.25540	22.11
Kollidon VA 64	96	0.25820	23.45
HPMC:Carbopol 974P NF (1:1)	0	0.20300	0.00
HPMC:Carbopol 974P NF (1:1)	24	0.23702	16.76
HPMC:Carbopol 974P NF (1:1)	48	0.24900	22.66
HPMC:Carbopol 974P NF (1:1)	72	0.25426	25.25
HPMC:Carbopol 974P NF (1:1)	96	0.26266	29.39

Formulation	Time (h)	Mass (g)	SI (%)
Carbopol 974P NF:Kollidon VA 64 (1:1)	0	0.20186	0.00
Carbopol 974P NF:Kollidon VA 64 (1:1)	24	0.23140	14.63
Carbopol 974P NF:Kollidon VA 64 (1:1)	48	0.24800	22.86
Carbopol 974P NF:Kollidon VA 64 (1:1)	72	0.25616	26.90
Carbopol 974P NF:Kollidon VA 64 (1:1)	96	0.26170	29.64
HPMC:Kollidon VA 64 (1:1)	0	0.19708	0.00
HPMC:Kollidon VA 64 (1:1)	24	0.22400	13.66
HPMC:Kollidon VA 64 (1:1)	48	0.23840	20.97
HPMC:Kollidon VA 64 (1:1)	72	0.24248	23.04
HPMC:Kollidon VA 64 (1:1)	96	0.24780	25.74

Table S2. Power-law model parameters for swelling profiles.

Formulation	k	n	R^2 log-linear	R^2 original SI scale	Interpretation
Carbopol 974P NF	1.426	0.664	0.958	0.945	Strongest time-dependent swelling among tested systems.
HPMC	6.072	0.194	0.984	0.983	Early hydration followed by a plateau-like trend.
Kollidon VA 64	3.886	0.400	0.990	0.984	Moderate time-dependent swelling.
HPMC:Carbopol 974P NF (1:1)	5.382	0.369	0.993	0.992	Moderate time-dependent swelling.
Carbopol 974P NF:Kollidon VA 64 (1:1)	2.945	0.514	0.980	0.974	Pronounced time-dependent swelling.
HPMC:Kollidon VA 64 (1:1)	3.366	0.452	0.963	0.957	Pronounced time-dependent swelling.

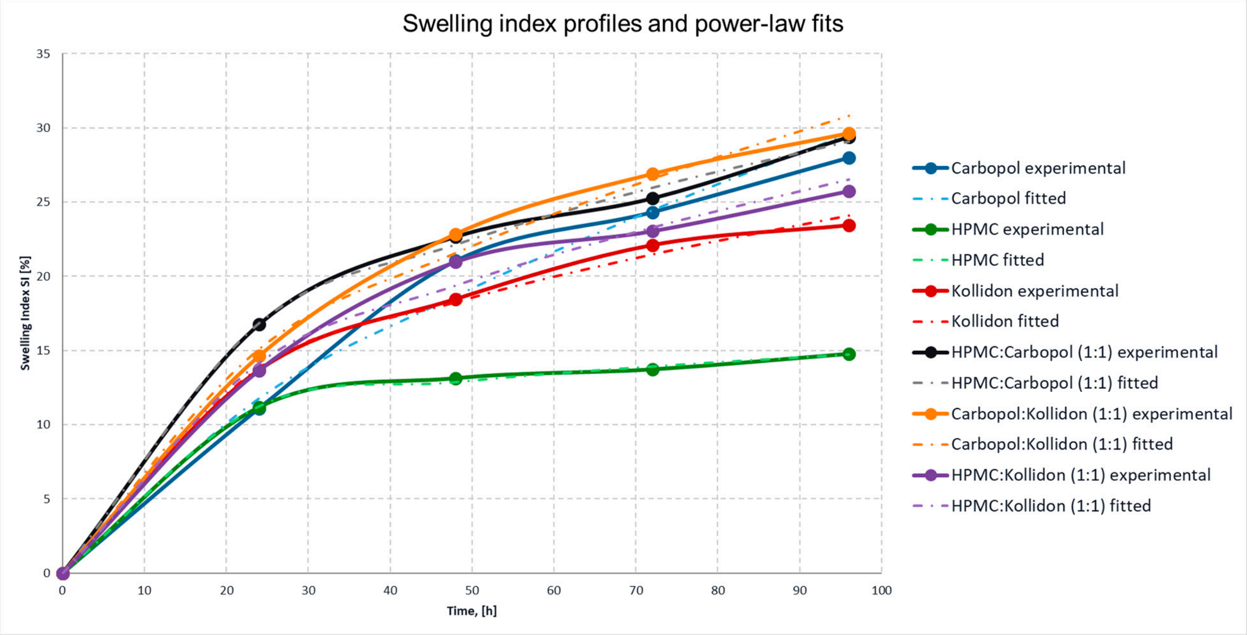

Figure S4. Points show calculated SI values based on mass changes; curves show the fitted empirical model: $SI_t = kt^n$.

Interpretation

The empirical power-law model provided a good descriptive fit for all investigated swelling profiles, with R^2 values of 0.958-0.993 for the log-linear regression. HPMC showed the lowest exponent (n = 0.194), which is consistent with rapid early hydration followed by a plateau-like trend. This agrees with the interpretation that a surface gel layer may limit further water penetration into the HPMC matrix. Carbopol 974P NF showed the highest exponent (n = 0.664), indicating the strongest time-dependent increase in swelling index over the investigated period. The HPMC:Carbopol blend showed a lower exponent (n = 0.369) than Carbopol alone, suggesting that the presence of HPMC moderated the swelling rate while still allowing substantial hydration. HPMC:Kollidon showed an intermediate exponent (n = 0.452), indicating pronounced time-dependent hydration; however, this should be interpreted together with the SDi2 observations showing reduced structural stability and erosion of Kollidon-containing matrices.

Overall, the fitted parameters support the conclusion that water uptake alone does not determine functional mucoadhesive performance. The most favorable behavior is associated with a balance between hydration, polymer-mucin interaction capacity, and matrix stability. The analysis is descriptive and should not be interpreted as a full mechanistic swelling model because it is based on a limited number of time points and summary mass values rather than replicate-level kinetic profiles.

Table S3. SDI2 images for polymers and their blends (1:1, w/w).

HPMC	Carbopol	Kollidon	HPMC:Kollidon	HPMC:Carbopol
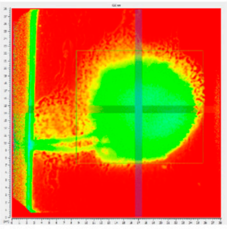<br>10 min	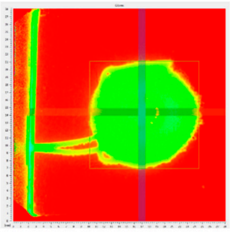<br>10 min	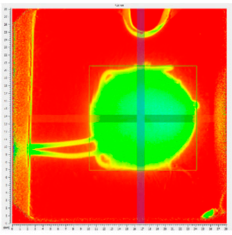<br>1 min	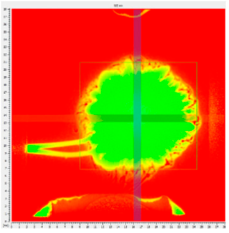<br>10 min	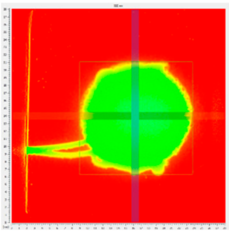<br>10 min
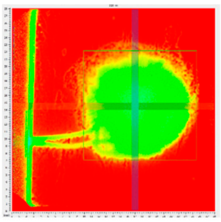<br>20 min	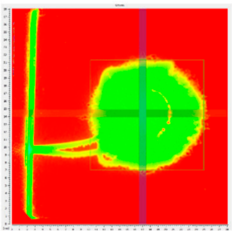<br>20 min	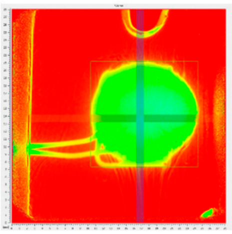<br>5min	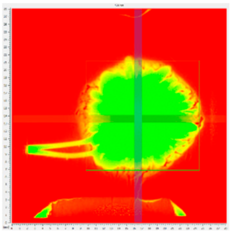<br>20 min	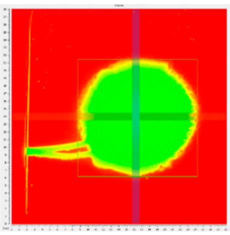<br>30 min
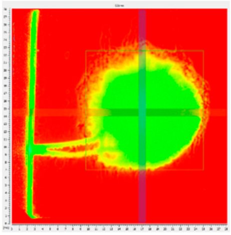<br>30 min	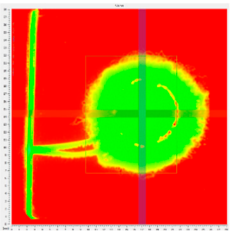<br>30 min	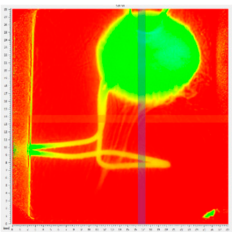<br>10 min	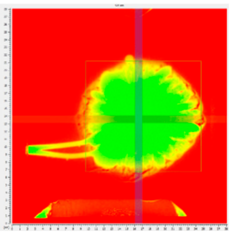<br>30 min	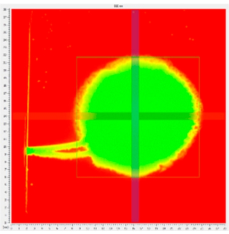<br>45 min
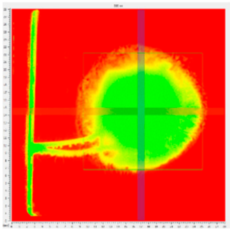<br>1 h	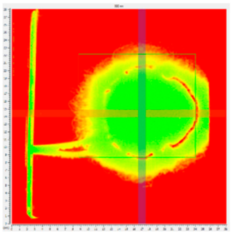<br>1h	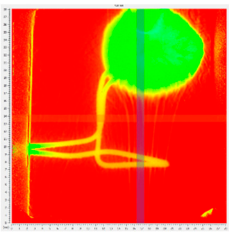<br>15 min	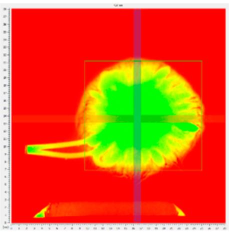<br>1h	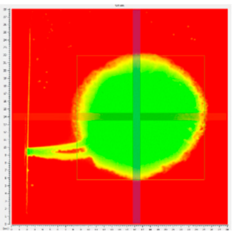<br>1 h
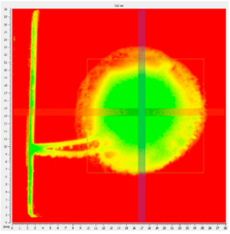<br>1h 30 min	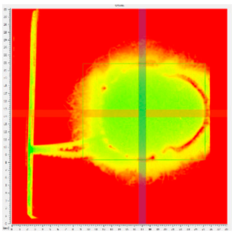<br>1h 30 min		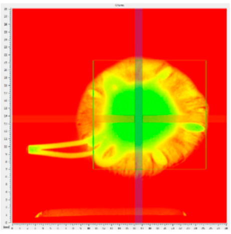<br>1h 30 min	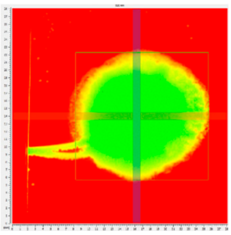<br>1h 30 min

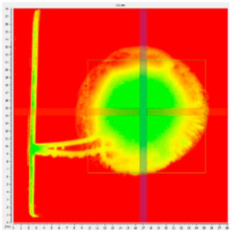
1h 45 min

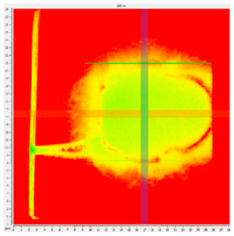
1h 45 min

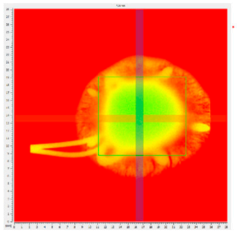
1h 45 min

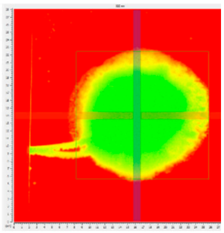
1h 45 min

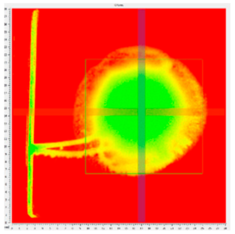
2 h

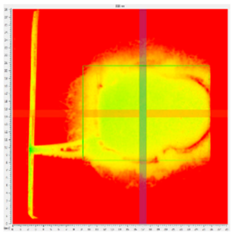
2h

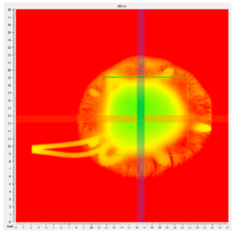
2h

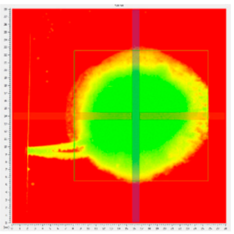
2h

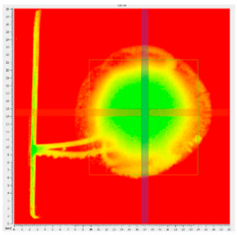
2h 30 min

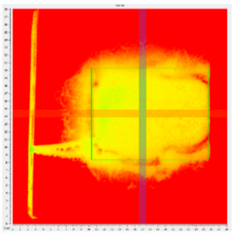
2h 30 min

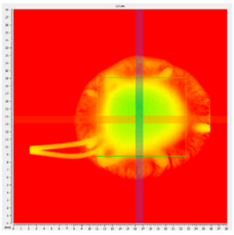
2h 30 min

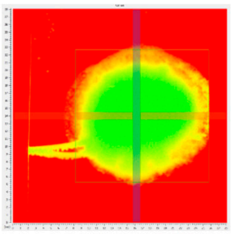
2h 30 min

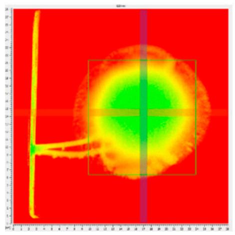
3h

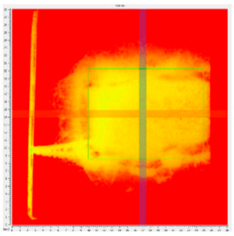
3h

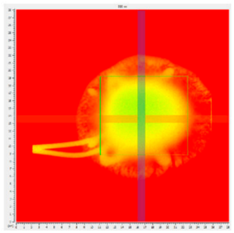
3h

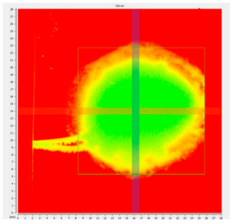
3h

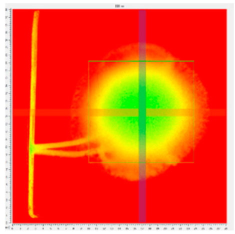
4h

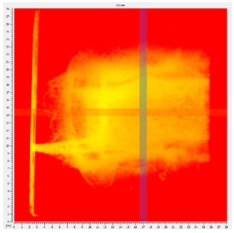
4h

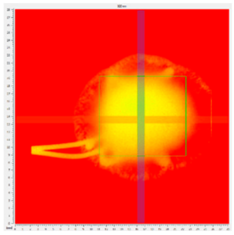
4h

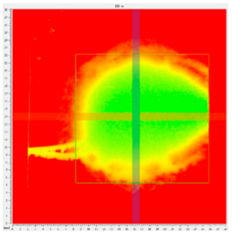
4h

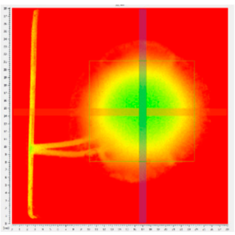
5h

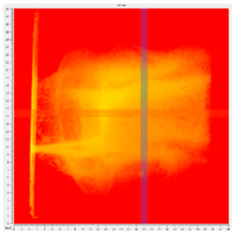
5h

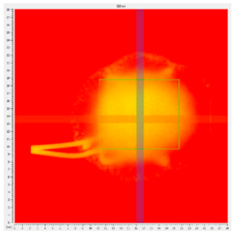
5h

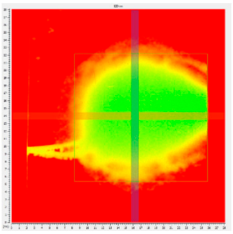
5h

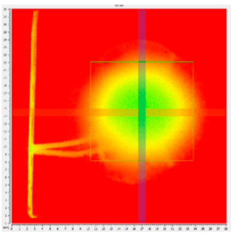
6h

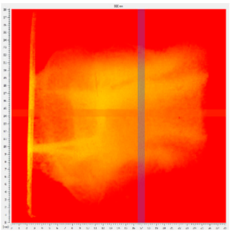
6h

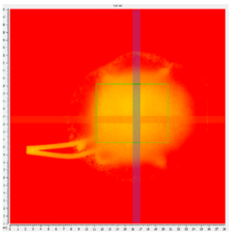
6h

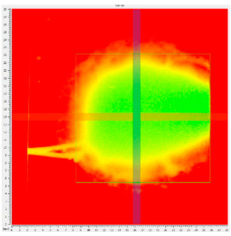
6h

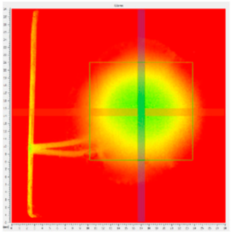
7h

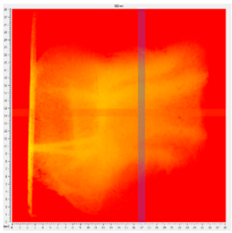
7h

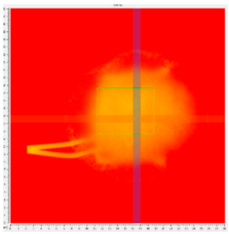
7h

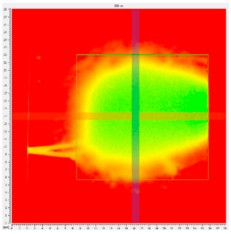
7h

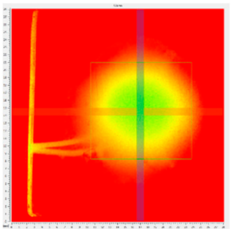
8h

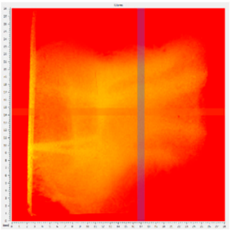
8h

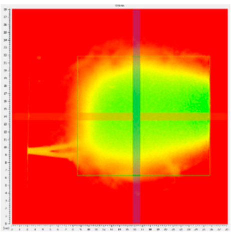
8h

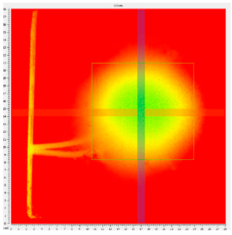
9h

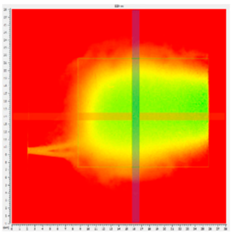
9h

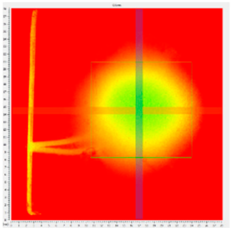
10h

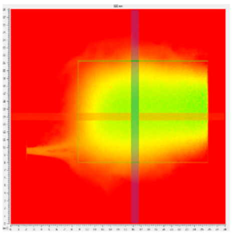
10h

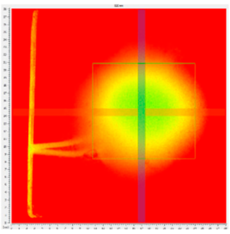
11h

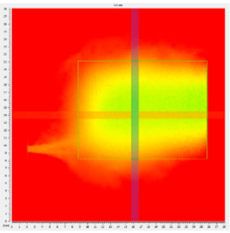
11h

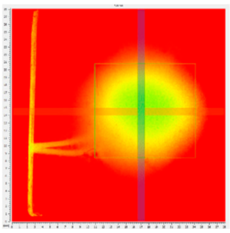

12h

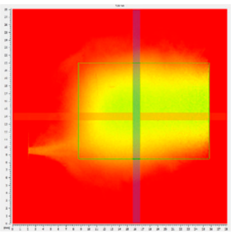

12h

4. **Supplementary Calculation: Pearson Correlation Between Surface Free Energy Components and Mucoadhesive Strength**

We show the calculation used to concerning the relationship between surface free energy (SFE) components and mucoadhesive strength. The calculation is descriptive, because only four directly comparable systems were available. Kollidon VA 64 was excluded because its mucoadhesive force could not be reliably quantified owing to rapid matrix disintegration during the adhesion test.

Data sources. SFE, polar component, and dispersive component values were taken from Table 4 of the manuscript. Mean mucoadhesive-force values were taken from the bar heights reported in Figure 4a of the manuscript for measurements performed in 2% (w/v) mucin solution. If the exact raw numerical means from the instrument output are later inserted, the same calculation shown below can be repeated directly.

For each SFE descriptor x and mucoadhesive force y, Pearson correlation was calculated as Eq.(S4):

$$r_{xy} = \frac{\sum_{i=1}^{n}(x_i - \bar{x})(y_i - \bar{y})}{\sqrt{\sum_{i=1}^{n}(x_i - \bar{x})^2 \sum_{i=1}^{n}(y_i - \bar{y})^2}} \qquad \text{(S4)}$$

where r_{xy} is the Pearson correlation coefficient, x_i denotes the value of a selected surface free energy descriptor, y_i denotes the corresponding mean mucoadhesive force, $\bar{x}$ and $\bar{y}$ are the mean values of x and y, respectively, and n is the number of analyzed polymer systems.

In this analysis, x_i represented total SFE, the polar SFE component (γ^p), or the dispersive SFE component (γ^d), whereas y_i represented the corresponding mean mucoadhesive force.
Because the analysis included only comparable systems for which mucoadhesive force could be reliably determined, the resulting correlation coefficients were interpreted descriptively.

Table S4 Input data used for the descriptive Pearson correlation analysis.

System	Mucoadhesive force, F (N)	Total SFE (mJ/m^2)	Polar component, γ^p (mJ/m^2)	Dispersive component, γ^d (mJ/m^2)	Data source
HPMC	0.78	12.0	11.3	0.7	Figure 4A; Table 4
Carbopol 974P NF	1.63	64.1	16.4	47.7	Figure 4A; Table 4
HPMC:Carbopol (1:1, w/w)	1.36	63.0	13.6	49.4	Figure 4A; Table 4
HPMC:Kollidon VA 64 (1:1, w/w)	0.60	60.7	12.8	47.9	Figure 4A; Table 4

**Kollidon VA 64 alone was omitted because the manuscript states that reliable detachment-force measurement was not possible for this material under the adhesion-test conditions.*

Table S5 Summary of Pearson correlations.

Correlation tested	n	$\bar{x}$	$\bar{y}$	*r*	Two-tailed p	Interpretation
γ^p vs F	4	13.5250	1.0925	**0.8434**	0.1566	Stronger positive descriptive association
γ^d vs F	4	36.4250	1.0925	0.4354	0.5646	Moderate/weak positive descriptive association
SFE vs F	4	49.9500	1.0925	0.4807	0.5193	Moderate/weak positive descriptive association

The p values are shown only for transparency. Because n = 4 and df = 2, the analysis should not be interpreted as inferential evidence of statistical significance. The conclusion is descriptive: within the comparable systems, the polar component showed the strongest positive association with mucoadhesive force

Using the mean mucoadhesive-force values reported in Figure 4a and the SFE components listed in Table 3, a descriptive Pearson correlation analysis was performed for the four comparable systems. The association with mucoadhesive force was strongest for the polar SFE component (r = 0.84), whereas weaker positive associations were obtained for the dispersive component (r = 0.44) and total SFE (r = 0.48). Because the analysis is based on only four systems and excludes neat Kollidon VA 64 owing to its rapid disintegration during adhesion testing, the correlations are interpreted descriptively. The result supports the conclusion that mucoadhesion is not governed by total SFE alone, but by the availability of polar/ionizable functional groups capable of specific polymer-mucin interactions and by the hydrated matrix maintaining structural integrity.
